# Recent advances in the combination of cellular therapy with stem cells and nanoparticles after a spinal cord injury

**DOI:** 10.3389/fneur.2023.1127878

**Published:** 2023-04-26

**Authors:** Elisa García, Samantha Sánchez-Noriega, Guadalupe González-Pacheco, Alejandro Naat González-Vázquez, Antonio Ibarra, Roxana Rodríguez-Barrera

**Affiliations:** Centro de Investigación en Ciencias de la Salud (CICSA), FCS, Universidad Anáhuac México Campus Norte, Huixquilucan de Degollado, CP, Mexico

**Keywords:** nanomaterials, nanoparticles, cellular therapy, spinal cord injury, combinatory therapy

## Abstract

**Background:**

Currently, combined therapies could help to reduce long-term sequelae of spinal cord injury (SCI); stem cell therapy at the site of injury in combination with other therapies has shown very promising results that can be transferred to the clinical field. Nanoparticles (NPs) are versatile technologies with applications to medical research for treatments of SCI since they could deliver therapeutic molecules to the target tissue and may help to reduce the side effects of non-targeted therapies. This article's purpose is to analyze and concisely describe the diverse cellular therapies in combination with NPs and their regenerative effect after SCI.

**Methods:**

We reviewed the literature related to combinatory therapy for motor impairment following SCI that has been published by Web of Science, Scopus, EBSCO host, and PubMed databases. The research covers the databases from 2001 to December 2022.

**Result:**

Animal models of SCI have shown that the combination of NPs plus stem cells has a positive impact on neuroprotection and neuroregeneration. Further research is required to better understand the effects and benefits of SCI on a clinical level; therefore, it is necessary to find and select the most effective molecules that are capable of exacerbating the neurorestorative effects of the different stem cells and then try them out on patients after SCI. On the other hand, we consider that synthetic polymers such as poly [lactic-co-glycolic acid] (PLGA) could be a candidate for the design of the first therapeutic strategy that combines NPs with stem cells in patients with SCI. The reasons for the selection are that PLGA has shown important advantages over other NPs, such as being biodegradable, having low toxicity levels, and high biocompatibility; In addition, researchers could control the release time and the biodegradation kinetics, and most importantly, it could be used as NMs on other clinical pathologies (12 studies on www.clinicaltrials.gov) and has been approved by the Federal Food, Drug, and Cosmetic Act (FDA).

**Conclusion:**

The use of cellular therapy and NPs may be a worthwhile alternative for SCI therapy; however, it is expected that the data obtained from interventions after SCI reflect an important variability of molecules combined with NPs. Therefore, it is necessary to properly define the limits of this research to be able to continue to work on the same line. Consequently, the selection of a specific therapeutic molecule and type of NPs plus stem cells are crucial to evaluate its application in clinical trials.

## 1. Introduction

Spinal cord injury (SCI) has detrimental effects on the spinal cord (SC), which results in the loss of motor, autonomic, and sensory functions with devastating consequences on the wellbeing and productivity of patients and their families. The pathophysiology of SCI is divided into primary and secondary injuries. Over time, the lesion progresses to a chronic phase, which prevents regeneration due to the development of the astroglial fibrous scar that surrounds the cystic cavities. The care of patients with SCI is a challenging task that requires the multidisciplinary collaboration of many scientists and specialists. Different therapies have been developed to reduce the damage related to the injury; however, a single treatment is not enough. Nowadays, combined therapies could help to reduce long-term sequelae; stem cell therapy at the site of injury is a well-studied option. Transplantation of mesenchymal stem cells (MSCs), neural stem cells (NSCs), and olfactory ensheathing cells (OECs) has been investigated as a potential therapy for SCI. It has been seen that MSCs can reduce the activation of the inflammatory response and promote functional recovery. NSCs are a source of astrocytes with beneficial functions, including the preservation of tissue integrity and neurotrophic support for neurons; finally, OECs are promising candidates for the promotion of neural repair as potential therapies for SCI. However, the neuroprotector and neuroregenerative effects are limited or depend on many variables; if these cells are combined with other therapies, very promising results can be obtained and transferred to the clinic. On the other hand, the use of nanoparticles (NPs) is a novel strategy used on diverse pathologies, due to their unique properties, are multifaceted nanotechnologies can offer efficient treatments because they have the ability to apply therapeutic molecules to the target tissue and therefore reduce the side effects by its high diffuse and compatibility. This article aimed to analyze and briefly describe the different types of cell therapies targeting MSCs, NSCs, and OECs individually and the advantages in combination with NPs as innovative treatments for their regenerative effects after SCI.

## 2. Incidence of spinal cord injury

Spinal cord injury is among the most worrying disabilities related to the nervous system. It is considered a global public health problem that affects patients physically, psychologically, and even socially. Furthermore, SCI refers to the damage to the SC as a result of some type of degenerative disease or due to severe trauma; vehicular accidents are usually the most frequent cause of SCI (1). According to the World Health Organization (WHO), it is estimated that each year there are between 40 and 80 new cases per million inhabitants worldwide of which 90% are of traumatic origin. Nevertheless, the number of cases of non-traumatic origin is constantly increasing as well (2, 3). This incidence is estimated at 10.5 new cases per 100,000 inhabitants per year. Singh et al. reported in 2014 that SCI has a higher prevalence in the United States of America with 906 cases per million inhabitants than in the Rhône-Alpes region, France and Helsinki, Finland, both with <280 cases per million population (4).

### 2.1. Pathophysiology of spinal cord injury

At present, a large number of economic resources are invested in different institutions around the world to carry out hundreds of investigations to study the pathophysiology and degenerative evolution of SCI and to learn more about the pathological mechanisms in order to develop new and more effective alternative therapies. This is a consequence of the worrying and increasing morbidity and mortality that this destructive neurological and pathological state entails (2).

In general terms, the pathophysiology of SCI is divided into an acute phase and a chronic phase. During its progression, a cascade of destructive events occurs from inflammatory processes, oxidative stress, cell apoptosis, and ischemia, which give place to the loss of locomotor and sensory functions (3). SCI triggers a series of biochemical and cellular events that eventually lead to the death of neurons, oligodendrocytes, astrocytes, and cell precursors. The main determinants of the severity of SCI are the extent of initial destruction (2, 3, 5).

Acute SCI commonly occurs from severe trauma to the spine where vertebral fractures or dislocations also occur. The primary injury appears immediately after the initial injury and is divided into two stages: Initially, it involves neural tissue damage, axonal connection interruption, glial membrane detachment, and hemorrhage. While clinical evidence suggests this causes a complete loss of functions, it has been observed that certain axonal connections remain during the primary SCI phase, demonstrating the existence of a state when there is only a partial lesion (2, 3, 6).

The secondary injury is triggered by the primary injury, causing other chemical and mechanical damages to the rachidial tissue. This produces neuronal excitotoxicity due to the high accumulation of calcium inside the cells increases the concentrations of reactive oxygen species and the release of glutamate. These events cause oxidative damage in proteins, phospholipids, and nucleic acids which can cause neurological dysfunction. Neuronal and glial damage include augmented cell permeability, edema, ion dysregulation, vascular damage, lipid peroxidation, apoptotic signaling, excitotoxicity, inflammation, demyelination, Wallerian degeneration, fibroglial scarring, and cyst formation (2, 3). Neuroinflammatory mechanisms could be induced by the events of secondary injury or on the other side the inflammatory response exacerbates it. For this reason, it is known that neuroinflammation after SCI can induce both beneficial and detrimental effects on locomotor function recovery; hence, it is necessary to characterize all the implications caused by inflammation to approach therapy research correctly (7).

The inflammatory response starts from the disruption of the blood–brain barrier (BBB), and the rupture of blood vessels causes hemorrhage into the spinal tissues, thus increasing the permeability of immune cells from the circulatory system to the lesion microenvironment, followed by monocyte, neutrophil, lymphocyte, and macrophage infiltration to the spine. The inflammatory cytokine output such as interleukin (IL)-1α, IL-1β, IL-6, and tumor necrosis factor (TNF-α), 6 to 12 h after injury, as a result of immune cells and anti-inflammatory cytokines presence, a neurological inflammatory process is given (3, 6, 8). During the acute phase (48 h after the lesion), vast inflammatory cells migrate into the exposed tissue and secrete cytokines (5). At this early phase, neutrophils reach their peak 24 h post-lesion. Afterward, the chronological arrival of inflammatory cells is characterized, in the subacute phase, by microglia peaking on day 7 and T cells peaking on day 9 after the lesion. Furthermore, at the late phase, a secondary injury cascade, starting 14 days after the injury, the peak of cellular inflammation takes place on day 60 post-lesion (characterized by the high prevalence of microglia) and it is still detectable for all three cell types over 180 days (9). Inflammation progresses through the action of pro-inflammatory cytokines, which include TNFα, gamma-interferon (IFNγ), CXC motif chemokine ligand 1 (CXCL1), IL-1, IL-12, IL-18, and the granulocyte–macrophage colony-stimulating factor (GMCSF), and it is solved with anti-inflammatory cytokines such as IL-4, IL-10, and IL-13 (10).

The presence of T helper cells (Th) in CNS is regulated by three specific points: subarachnoid vessels, choroid plexus and parenchyma, and post-capillary venules. CD4+ T cells might also become tissue-resident after inflammatory episodes (11). These cells modulate how innate and adaptive immune cells function and are recruited through the secretion of anti-inflammatory cytokines and growth factors. One crucial feature of T cells is the immunological synapse, which is the connection that allows an adaptive immune response to mediate injury repair and then differentiates to variable phenotypes of T cells according to the cytokines that are released, except for Th1 and Th2 subsets (12). The acute lesion is usually distinguished by the presence of Th1 subsets that control the discharge of pro-inflammatory cytokines, such as IL-2, IL-12, and IFNγ, therefore resulting in demyelination and parenchyma destruction, although their possible beneficial effects are still under debate. Prior to that, at the subacute phase, Th2, Treg, and Th17 prevalence can be identified due to the release of IL-4, IL-10, IL-17, and IL-23 cytokines, protecting the spine from autoimmune effects. If the microenvironment characteristics are optimal and the susceptibility of the individual is appropriate, recovery could be enhanced during this phase by reducing neuroinflammation (11). However, several articles show that different Th phenotypes can mediate immune-mediated damage, Exacerbated inflammatory response or even generate autoimmune CNS inflammation (9, 13). In addition, inflammatory conditions allow the predominance of T-cell phenotypes, such as Th1 and Th17 (14). Even though they mainly secrete a profile of pro-inflammatory cytokines, they also contribute to lesion repair by different pathways: Reactive astrocytes help to the restoration of BBB by inducing endothelial tight junction repair, cleaning cellular debris, and reducing excitotoxicity (15). Reactive astrocytes form a dense glial scar at the edge of the lesion surrounding a central lesion composed of proteoglycans and activated phagocytic microglia/macrophages. These cells have a constant cross-talk interaction with phagocytic cells by the release of different profiles of cytokines and chemokines (16). The formation of this scar limits the spread of necrotic, apoptotic, and infiltrating inflammatory cells, inhibiting neuroinflammation (7). Furthermore, negatively, the formation of glial scar interrupts axonal regeneration (15). To counteract all these harmful effects, strategies have been designed for therapeutic biomaterials to control neuroinflammation.

Now, different drugs regulate or inhibit different mechanisms involved in the inflammatory response; however, the direct effect is not possible and that limited the recovery after SCI and has a secondary consequence that depends on the type of drug administered. A viable strategy for solving it is nanomedicine, which has increased its importance and presence in different investigations in recent years. This has a fundamental role in the design of new therapeutic strategies in pharmacology, such as the controlled kinetic release and bioavailability of active ingredients, managing to increase the effectiveness of already established treatments, and reducing adverse effects. In the technological branch, nanomaterials such as NPs and nanofibers are being developed to serve as a replacement for the neural tissue affected or lost in SCI, as well as nanopharmaceuticals that manages to integrate the tissue to its normal state (17–19).

## 3. Nanomaterials for neuroprotection and neuroregenerative processes

In the past few years, active explorative studies had analyzed the relevance of nanomaterials (NMs) as a treatment for neurodegeneration and the processes they imply. NMs and the different applications they have been given make possible drug delivery on SC or between a combination strategy with cellular therapy by promoting its differentiation or action intending to promote neuroregeneration. The mentioned approaches seek to become possible solutions to trespass the BBB (20).

Nanomaterials are materials with nanoscale dimensions, whose proportions vary from 1 to 100 nm. These materials, in contrast to other bulk molecules, have singularities that distinguish them. In recent decades, NMs gained insight as environmental-safe, lightweight, pH labile, and easily disintegrated materials (20, 21). NMs are known to have a promising role in the intervention of neuronal injury (21).

Nanomaterials could be the answer to solving therapeutic issues because of their particularities such as their size. They offer the possibility of employing them as nano-carriers specifically targeted to the site of injury for enhancing the bioavailability of drugs and broadening their time in circulation (22). In addition, NM-formed NPs are able to restore axonal regeneration with regard to promoting signal pathways on neural cells or stem cells applied, which could reinstitute conduction in SCI through progressive axonal re-growth (23). Scaffolds constructed upon NMs aid in microenvironment recreation, according to its fundamental nature (24).

Not long ago, diverse research works were dedicated to identifying NMs as a treatment for SCI. By their nano-size ranges, the neuroprotective drugs compacted by NMs increase their availability specifically on the SC site of injury. Nanomaterial-mediated drug delivery has exposed relevant therapeutic advances, showing results like reduced neuronal damage which allows motor recovery. In addition, this drug delivery system has been engineered and utilized as dendrimers, nanocapsules, silica NPs (SNPs), carbon nanotubes, and graphene sheets to prevent further neurodegeneration after SCI (25). The study of nanostructured drugs has been sectioned in two: (1) drug encapsulation within the nanostructure during the time of synthesis and its coupling with conjugation reactions and (2) promotion under general development, growth, or differentiation of stem cells in the treatment of neurological diseases (26).

## 4. Nanoparticles on spinal cord injury

Multiple variants of NPs have been under trial for the treatment of SCI. The components of NPs vary from metals or ceramics (gold, silver, iron oxide, cadmium, and zinc) (27–29), polymers [poly(lactic-co-glycolic acid (PLGA), and polycaprolactone] (30, 31), liposomes (32, 33), exosomes (34, 35), carbon-based NPs (CNPs), SNPs (36) and numerous other types (37–39). The various existing types of NPs have intrinsic advantages and disadvantages; therefore, a meticulous investigation should be pursued before selecting a particular type of NPs regarding the specific requirements of the study. The relevance of this rigorous selection is a consequence of the potential problems associated with BBB permeability and the complex organization of the CNS when applying NPs in the SC. The different properties of NP charge and surface are important parameters to take into consideration for SC applications (40). Positively charged variants have been tested within experimental models of SCI, being more efficiently internalized into cells in comparison with neutral and negatively charged NPs (41). In addition, positively charged NPs have a greater absorption into the liver when they are circulated in the bloodstream (42). Concerning the uptake of negatively charged NPs, it has been shown to be more agile when penetrating tissues than positively charged particles, while the positively charged particles are taken up more rapidly by proliferating cells (43). The specificity of NPs' surface charge depends on the purpose of their application, and the characteristics of the surface charge are capable of creating unique interactions with cells, leading to more desirable outcomes following recovery in SCI.

Nowadays, nanotechnology as a therapeutic approach for SCI has gained inordinate progress using different types of NPs for drug delivery, on Table 1 resumed the principal advantages.

**Table 1:** Different types of nanoparticles used on spinal cord injury.

**Nanoparticles**	**Synthesis characteristics**	**Advantages**	**Results after SCI**	**References**
Iron oxide	Superparamagnetic iron oxide NPs (IONPs) sizes vary from 5 to 300 nm.	Permit for cell recognition following cellular implantation into the SC.	Cells with internalized IONPs can be tracked *in vivo* using magnetic resonance imaging (MRI). Iron oxide-labeled MSCs were targeted after SCI using magnetic fields.	(44, 45)
Noble metal/nanorods	Polyethylene glicol (PEG) is mostly applied for the efficiency of NPs circulation and tissue diffusion because of their surface, facilitating the penetration of NPs into the CNS. This is allowed mainly by increasing their stability on circulation, decreased capacity to form conglomerates and their linkage with plasm and proteins further and high dissolution degree by hydrophilic character.	Chemistry modification techniques are readily done to PEG in order to add other particles onto their surface.	PEG coated on the surface of gold NPs (GNPs) has shown to enhance recovery after SC injuries. NPs covered with gold tissue engineering scaffolds have already demonstrated *in vitro* to promote axonal growth in neurons. *In vitro* neurons with gold nanorods after being exposed to radiation with a 780-nm laser displayed higher axonal and dendrite regeneration in comparison with control models in absence of radiation, as a result, they could be used after SCI with restoring effects. For the 15-nm gold NPs are, as well, employed as adjuvants for protein immunization, improving their effects, besides increasing neurite outgrowth.	(28, 46–49)
Quantum dots	Quantum dots (QD's) are crystalline semiconductors, which structure is regularly compound by a semiconducting material inside (usually cadmium or zinc) added to a stabilizing exterior. Their sizes range from 1 to 10 nanometers.	QDs mainly function as probes that display high fluorescent intensity, have wide absorption and narrow emission spectra, and are photobleaching resistant, on account of their size-induced quantum confinement. Unfavorably, experimental cultures and NS models carried out in a living organism have shown toxicity, which confines their current use for research purposes solely, mostly for cell imaging.	QD's performance was tested within a chick embryo SC model, and their mobility was tracked during development by fluorescence techniques. The implementation of ZnO QD's is mainly for neuron recount in SC murine models. Moreover, immunohistochemistry has been applied to QD's in reason of tracking astrocytes in SC.	(50–52)
Polymer NPs	Polymer NPs have variable uses in the SC and most importantly for delivery. The proportions of NPs can be broad from 50 to 1,000 nm, and also mainly present a spherical shape.	Synthetic polymers such as PLGA, (PLLA), and polycyanoacrylate are considered natural polymers that have demonstrated low toxicity levels in the CNS, allowing their role in the local delivery of therapies to the SC. Between these, the PLGA is approved by the FDA and European Medicines Agency (EMA). FA–GCNPs in various drug delivery systems, leading PLGA-based NPs to a convenient situation for clinical trials.	Biodegradable polymers such as PLGA have the capability of drug release from delivery systems and in diverse studies could be used as tissue scaffolds. PLGA offer the possibility to prevent drug depletion and improve their cohesion. In pursuance of confirming the efficacy of PLGA in drug delivery system, Lowry et al., encapsulated sonic hedgehog (Shh) into PLGA microspheres and examined whether the release of Shh by these microspheres at SCI is able to aid in the regenerative process by inducing prominently oligodendrocyte and neuron proliferation. They showed that biodegradable PLGA microspheres function as minimally invasive tools for the delivery of therapeutic agents to the injury site. In a different study, PLGA NPs approach was for local delivery of flavopiridol (cyclin-dependent kinase CDK inhibitor) to injury site, cavity formation and neural loss was decreased, additionally, it enhanced neuronal regeneration.	(29, 31, 53–56)
Dendrimer NPs	Dendrimers display a vastly ramified 3D architecture with an initiator core. Repeating units conform the inside, and various active terminal groups cover the surface. Dendrimer sizes applied in the CNS vary from 5 to 200 nm.	Dendrimers NPs are capable of delivering therapeutics for regeneration after SCI.	Following of lateral hemisection to the SC model in rats SCI, dendrimers were used for methylprednisolone delivery following SCI injection of these NPs improved BBB (standardized open-field locomotor scale for recovery) scoring to 4 weeks in contrast with controls.	(57–59)
Lipid NPs	Lipid NPs used in the CNS are usually liposomes, conformed by phospholipids or amphipathic lipids, which size varies from 20 to 100 nm.	The lipid NPs can transport lipophilic drugs due to their biocompatibility, biodegradability, and the low toxicity shown in *in vivo* models These are considered a novel nanocarrier for drug administration because they can transfer the BBB.	After SCI in a study, the use of liposomes decreased macrophage migration inhibitory factor, reducing secondary injury after SC on a contusion model in rats.	(32, 33, 60)
Carbon-based NPs/CNPs	CNPs applied in the SC include fullerenes and carbon nanotubes as single-wall (0.4–3 nm diameter) or multi-wall (1.4–100 nm diameter).	Fullerenes have shown neuroprotective features *in vivo* as a result of their chemical ability to neutralize free radicals.	CNPs have been under trial within multiple animal models of CNS injury. Multi-wall with Nogo-66 receptor siRNA, RhoA siRNA, and brain-derived neurotrophic factor DNA, has been probed after SCI in rats, were these presented elevated BBB scores at 8 weeks. Single-wall improved BBB scores of SC-injured rats 7 weeks following SCI. Fullerenes have also been applied to murine models of amyotrophic lateral sclerosis and experimental autoimmune encephalomyelitis. Regardless of its advantages, toxicity concerns remain and must be cleared to their approval for clinical use.	(39, 61–63)
SNPs	SNPs, whose proportions vary from 5 to 1,000 nm, can be assembled mesoporous (pore size 2–50 nm), providing them a wide exterior area, which enables them to load drugs extensively within the porous structure, advantage that has been applied on SC.	SNPs initial effect seems to depend on the concentration (higher concentrations are associated with cell toxicity and death).	SNPs supplied to an *ex vivo* guinea pig SC model, demonstrated the ability to seal neuronal membranes following SCI, however, it is necessary to prove their biocompability.	(36, 37, 56)

## 5. Use of cellular therapy and nanoparticles after SCI

Spinal cord injury implies that SC microenvironment changes in the early weeks post-injury, such as scar formation; therefore, combined therapies are needed. Animal model studies have shown that stem cell grafts are a potentially effective approach for SC regeneration by substituting necrotic nerve cells with differentiated MSCs, NSCs, and OECs; novel supporting cell transplants for remyelination, re-growth, and connection of the injured axons; and provide a protective environment for cells when transplanted into the injury site to prevent further harm by releasing protective substances such as growth factors, decreasing toxins such as free radicals, and preventing the spreading of the injury by reducing inflammation posterior to injury (64).

## 6. Recent advances in the combination of cellular therapy with stem cells and nanoparticles after a spinal cord injury

### 6.1. Mesenchymal stem cells and nanoparticles

Mesenchymal stem cells are an attractive approach for cellular therapy due to their self-renewal ability and differentiation to multiple variable mesenchymal lineages. As multipotent cells, MSCs are able to differentiate into other mesodermal tissues such as bone, cartilage, and cardiac muscle. Immunomodulatory properties and paracrine mechanisms of MSC benefits have been described in multiple studies (65, 66). MSCs have favorable effects by stem cell therapy after SCI, as a result of the advantages listed: (1) simple isolation and cryopreservation, (2) preserve regenerative ability and self-renew ability even at −80°C, (3) elevated proliferation rate and potential of multilineage differentiation, and 4) decreased the immunoreactivity and graft-vs.-host reaction of transplanted allogeneic MSCs (66, 67).

The principal sources of MSCs are bone marrow, umbilical cord blood, adipose tissue, and peripheral blood, even though they can be obtained from a variable range of tissues. MSCs are a viable option for lessening the negative pathophysiological effects of SCI (65). In regard to recovery after SCI, therapeutic methods mainly focus on achieving axon re-growth, inhibition of apoptosis, and substitution of damaged cells with oligodendrocytes in favor of axon remyelination (68). When MSCs are administered to the injury site following SCI, they produce and release different growth factors, including brain-derived neurotrophic factor (BDNF), glial derivate neurotrophic factor (GDNF), vascular endothelial growth factor (VEGF), insulin-like growth factor (IGF), fibroblast growth factor (FGF-2), and transforming growth factor (TGFβ), the molecules that promote axonal regeneration. In addition, MSCs downregulate apoptosis by reducing levels of apoptotic molecules and increasing the survival molecules in SCI animal models. Another important effect of these cells on cytokines is an increase in serum IL-10 and a decrease in tumor TNFα, which plays a relevant role in T-cell transitions from pro-inflammatory Th1 to anti-inflammatory Th2 phenotype, as well as macrophage phenotype transition from M1 (immune surveillance) to M2 (downregulating immune response). Through these mechanisms, MSCs enhance the survival and development of neurons and axonal regeneration (69, 70).

Stem cell transplantation is receiving increased attention as a therapeutic approach for the treatment of neurological diseases (71, 72). At the time of writing this article, there were 52,055 clinical trials involving neurological diseases and 743 involving stem cell-based therapy for neurological diseases. After SCI, only 60 studies included stem cell-based therapy and only six studies used NPs and stem cells on other pathologies (www.clinicaltrials.gov, accessed on 8 December 2022). Although a limited research area exists for stem cells' potentiated effect with NPs, MSCs have an immunomodulatory effect; however, none of these cells have produced more than a partial recovery of function, being an active area of research.

The therapeutic strategies that combination with NPs could potentially exacerbate the neurorestorative effect of MSCs, based on these foundations in 2015, Tukmachev et al. exposed that superparamagnetic IONPs can promote the taking in of MSCs at the injury site in a rat model of SCI (45). In the same context, Vishwakarma et al. developed a novel gadolinium-SPIO (Gd-SPIO) that acts like magnetic NPs with an elevated level of biocompatibility and potentially acquires a better contrast in MRI. As a result, it was proposed that the transplantation of neuronal cells labeled with Gd-SPIONPs could be an excellent approach for achieving high-contrast imaging and non-intrusive cell trapping at specific sites while remaining unaffected by inhibitory molecules. In a different study, magnetically labeled MSCs were introduced into the subarachnoid space in both the magnet and non-magnet groups. MSCs agglomerations were detected superficial to the SCI in the magnet group; on the contrary, few MSCs were observed in the non-magnet group. The hindlimb motor function of the magnet group displayed a significant improvement in comparison with the non-magnet groups (73). Furthermore, this combined therapeutic approach for regeneration after SCI is capable of potentiating the use of tissue engineering strategies to effectively bridge the injury (74). As regards, anti-inflammatory, anti-oxidative, and anti-excitotoxicity effects of ferulic acid (FA) and glycol chitosan (GC), both can be contemplated as natural neuroprotective compounds (75, 76). FA is capable of absorbing a hydrogen atom to form a phenoxy radical by the phenolic hydroxyl group; as a result, it protects cells from oxidative stress (76). Apart from that, the primary amines of GC might have a relevant role in neuroprotection. Wu et al. produced hydrophobic self-assembled FA-GCNPs by chemical conjugation, which displayed neuroprotective effects of FA–GCNPs following SCI. Systemic administration of these NPs promoted neuron cells and axons survival at the injury site and reduced the number of macrophages and activated astrocytes. Hence, these neuroprotective effects led to significant recovery after SCI (77).

The regenerative efficacy of IONPs-treated human MSCs was evaluated using exosome-mimetic nanovesicles (NV-IOPs) in a SCI model, with the results showing an increased amount of therapeutic growth factors associated with IONPs, which were slowly ionized to iron ions for the activation of JNK and c-Jun signaling cascades in human NSCs. *In vivo* systemic administration of these with magnetic guidance upregulated their number at the injury site. In addition, NV-IONP agglomeration promoted angiogenesis, generated anti-inflammatory and anti-apoptotic effects in SCI, and therefore had beneficial effects on SC function (78).

The principal focus of experimental models of SCI is stem cell transplantation into injured SC for motor recovery; however, bladder dysfunction investigation is almost none. However, Lee et al. investigated the effect of B10 human MSCs grafts into the bladder wall of SCI rats to prove their capability of collagen deposit inhibition and cystometric parameter improvement. Consequently, the SCI rats decreased their weights and collagen deposition, and furthermore, these cells differentiated into smooth muscle cells. The transplanted B10 cells helped to prevent bladder fibrosis and bladder dysfunction in the rat SCI model. Therefore, MSCs-based cell transplantation is a potential therapeutic approach for bladder dysfunction in patients with SCI (79).

Human NSCs are derived from induced pluripotent stem cells (iPSC-NSC), human MSCs, and a pH-responsive polyacetal–curcumin nanoconjugate (PA-C), which promote the sustained secretion of curcumin delivered into the intrathecal space in SCI contusion model in rats with stem cell transplantation. As a consequence, the locomotor function was improved (BBB scale), scars had reduced in size, and β-III tubulin-positive axons, motoneurons, and myelinated tracts were preserved. PA-C therapy promoted microglia into an anti-inflammatory phenotype (80). These studies advocate that MSC combination with other therapies is possible in favor of boosting their therapeutic potential. For example, another cell therapy is NSCs.

### 6.2. Neural stem cells and nanoparticles

These cells are multipotent cells with a high capacity for self-renewal and multidirectional differentiation potential to separate themselves into mature neurons and glial cell phenotypes. This provides a promising strategy, which could restore neuron loss after injury or in disease states. However, treatments with NSCs present challenges in terms of their regulation and evaluation of outcomes, including survival, distribution, differentiation, and integration. Long-term therapies generally focus on secondary damage alterations such as neuroinflammation and maladaptive plasticity, aiming at preserving the tissue and multiplying the production of exogenous NSCs to increase regeneration (26, 81). The combination of nanotechnology with stem cells is continuously developing and has shown that it can favor clinical and therapeutic diagnosis in various CNS diseases. NPs are innovative components for regenerative medicine due to their physicochemical characteristics (26, 82); they can be made of organic, inorganic, or composite-based materials and can be combined with different polymers to improve their hydrophilicity, biocompatibility, bioavailability, targeting capacity, and approach efficacy that can reduce dose-related and drug release at the site of injury (29).

GNPs provide a very effective method for introducing substances into cells and are characterized as being non-toxic, non-immunogenic, and biocompatible, which makes them ideal resources to be applied in biomedicine (83). The GNPs are distributed as negatively charged or positively charged. After SCI, GNPs positively increase neuronal growth when incorporated into embryonic cells or NSCs when applied on rats with SCI, promoting neuroregeneration and motor recovery function, thus suggesting that transplantation may be an effective therapy for injury repair (84).

Chitosan NPs (ChNPs) and valproic acid (VANPs) have shown efficacy as protective agents in SCI treatment. A study in 2021 found that ChNPs treatment with VANPs benefited tissue regeneration and locomotor function after SCI, significantly improved NSC proliferation and secretion levels of neurotrophic factors, promoted the outgrowth of neurite since it stimulated overexpression of microtubule-associated protein, and decreased IL-1β, IL-6, and TNF-α expression (85).

Chitosan NPs have chemical characteristics that are determined by two variables: molecular weight and the degree of acetylation (DA). Previous research has shown that DA plays a key role in the incorporation of artificial membranes, the sealing of mammalian membranes, and, finally, the restoration of neurophysiological function *ex vivo* (56).

Poly[lactic-co-glycolic acid] is a biodegradable polymer approved by the US FDA and the EMA. It has drawn attention due to its excellent biocompatibility and biodegradability, as well as its predictable biodegradation kinetics. Due to these characteristics, it has been used as a nanocarrier, has low toxicity and immunogenicity, and allows prolonged drug release. PLGA-encapsulated methylprednisolone has been used in SCI, and it has demonstrated a decrease in secondary injuries related to inflammation as well as a significant regenerative outcome 7 days post-injury (30). Another study has used electrospun nanofibers of PLGA and PEG, which enhanced the adhesion and proliferation of NSC derived from induced pluripotent stem cells (iPS). iPS derived from NSCs proved greater differentiation potential on the exterior of PLGA/PEG biomaterials. Furthermore, scaffold strategies combined with iPSC-NPC therapy have been demonstrated to improve functional SC rehabilitation after transection (86).

Neuroregeneration through NSCs in combination with NPs could provide promising therapies to repair and/or regenerate the damage caused by neurodegenerative diseases; likewise, these therapies may emerge as alternatives in the diagnosis of some CNS disorders.

In SCI, the use of NPs can increase the survival rate, decrease the diffusion of stem cells after transplantation, elevate the rate of differentiation into functional neurons, and direct the growth of stem cells. NPs and stem cells can be complementary therapeutic options.

### 6.3. Olfactory ensheathing cells and nanoparticles

Olfactory ensheathing cells are a resident of glial cells which are found in the peripheral nervous system (PNS) and CNS and are mostly observed in the central olfactory bulb (OB) and the nasal olfactory mucosa (87). They come with the surface of olfactory nerve fibroblasts, in order to hold the bundles of olfactory nerve fibers from the nasal mucosa for permitting synapsis in the OB (88). A study has shown that OB transplants can be differentiated for generating connections between the periphery and brain (87).

Brain-derived neurotrophic factor, GDNF, and NGF are expressed for OECs, and these are important for the propagation and guidance of axons, displaying similar features with astrocytes and Schwann cells (89, 90). The neurotrophic factors are able to protect neurons as a result of their capacity to inhibit the formation of scars and promote axonal re-establishment (91). They also are crucial for neural regeneration in reason of their capacity to proliferate and migrate from PNS and CNS. Recent studies have shown that extracellular vehicles (EVs) with OECs regulate cell–cell communication, viability, and toxicity (92).

Additional studies are needed to know more about the effect of the combining OECs and NPS ChABC-loaded PLGA at the injured site 1 week following the contusion, demonstrating myelin in the group with NPs ChABC-loaded PLGA and a significant therapeutic effect on functional recovery (93). Other outcomes demonstrate a function for human OEC-derived EVs in NSC proliferation and oxidative stress-induced neuronal toxicity model (94).

Fan et al. showed that isolated exosomes by the PEG-based method are a critical role in immunomodulation and could be engulfed by microglia and polarized anti-inflammatory response after SCI. The disadvantage is that the categorized EVs may perhaps trespass on other cell membranes, causing the results to be misinterpreted. Further studies are needed with regard to giving greater evidence about the effectiveness of this combinatory therapy with OECs and NPs (34).

## 7. Limitations and future prospects

Animal models of SCI have demonstrated that the combination of NPs plus stem cells has a positive impact on neuroprotection and neuroregeneration. Additional studies are needed for a better understanding of the effects and profits of SCI on a clinical level; therefore, it is necessary to find and select the most effective molecules that are capable of exacerbating the neurorestorative effects of the different stem cells and then try them out on patients after SCI. On the other hand, we consider that PLGA NPs could be the first therapeutic strategy that combines NPs with stem cells that could be used on patients with SCI because these NPs have shown important advantages compared to other NPs that have been previously approved by the FDA. These specific NPs are biodegradable and have shown low toxicity levels and high biocompatibility. In addition, researchers could control the release time and the biodegradation kinetics, and most importantly, it could be used as NMs on other clinical pathologies (12 studies on www.clinicaltrials.gov).

## 8. Conclusion

The use of cellular therapy and NPs can be an attractive strategy for SCI therapy; nevertheless, the data obtained from interventions after SCI will reflect an important variability of molecules used on NPs. Therefore, it is necessary to properly define the limits of this research to be able to continue to work on the same line. Consequently, the selection of a specific therapeutic molecule and the type of NPs plus stem cells is crucial to evaluate the application in clinical trials.

## Author contributions

EG: conceptualization, formal analysis, investigation, methodology, project administration, writing the original draft, and reviewing and editing. SS-N: data curation, investigation, methodology, and reviewing language and editing. GG-P: data curation, investigation, methodology, writing the original draft, and reviewing language and editing. AG-V: data curation, investigation, methodology, and writing the original draft. AI: supporting and project management supervision. RR-B: conceptualization, formal analysis, investigation, methodology, project administration, writing the original draft, reviewing and editing, and project administration management (main). All authors contributed to the article and approved the submitted version.
